# Editorial: The role of glycans in infectious disease, Volume II

**DOI:** 10.3389/fmicb.2022.1074656

**Published:** 2022-11-04

**Authors:** Iván Martínez-Duncker, Héctor M. Mora-Montes, Gerardo R. Vasta, Fabrizio Chiodo

**Affiliations:** ^1^Laboratorio de Glicobiología Humana y Diagnóstico Molecular, Centro de Investigación en Dinámica Celular, Instituto de Investigación en Ciencias Básicas y Aplicadas, Universidad Autónoma del Estado de Morelos, Cuernavaca, Mexico; ^2^División de Ciencias Naturales y Exactas, Departamento de Biología, Universidad de Guanajuato, Guanajuato, Mexico; ^3^Department of Microbiology and Immunology, University of Maryland School of Medicine, Institute of Marine and Environmental Technology, Baltimore, MD, United States; ^4^Department of Molecular Cell Biology and Immunology, Amsterdam UMC, Vrije Universiteit Amsterdam, Amsterdam, Netherlands; ^5^Institute of Biomolecular Chemistry, National Research Council (CNR) Pozzuoli Napoli, Pozzuoli Napoli, Italy

**Keywords:** ABO, *Escherichia coli*, glycosphingolipids, glycans, infections, COVID, virus

Volume 2 of “*The role of glycans in infectious disease*” Research Topic offers a continuity of research articles highlighting the evolving relevance of the glycocode at the interphase of pathogens and human disease, where it modulates the dynamics of bacterial, viral and fungal infections through finely tuned mechanisms. In this sense, mucosal surfaces are probably the sites where the most complex host-microbe interactions take place. In an original research article, Suwandi et al. characterize the role of the beta-1,4-N-acetylgalactosaminyltransferase enzyme (B4galnt2) in modulating infections by *Citrobacter rodentium*, a murine model pathogen for human enteropathogenic and enterohemorrhagic *Escherichia coli. Escherichia coli* is a gram-negative bacteria that can usually colonizes the human gut, but can cause intestinal or extraintestinal infections, including severe invasive disease such as bacteremia and sepsis. In this regard, *E. coli* is the most common cause of bacteremia in high-income countries and is a leading cause of meningitis in neonates (Bonten et al., 2021). Through the use of adhesion assays based on intestinal epithelial organoid-derived monolayers from B4galnt2^−/−^ and B4galnt2^+/+^ mice the authors show that lack of this enzyme causes increased *C. rodentium* adherence, promoting increased inflammation and less proficiency in pathogen clearance by the host. The enhanced pathogen adhesion is dependent on the interaction of type 1 fimbriae and host mannosylated glycans that are increased as a result of deficient B4galnt2, as revealed by increased staining of the *Galanthus nivalis* lectin. These novel findings contribute to establish a more precise role of this enzyme in modulating host-pathogen interactions, but also in the establishment of gut microbiota.

Cell surface glycosphingolipids are involved in a variety of cellular processes, including proliferation, apoptosis and migration, playing key roles also in inflammation, infection and cancer progression (Schnaar et al., 2022). Furthermore, although most pathogen receptors have been identified as host cell surface glycoproteins, evolutionary pressure has also shaped glycosphingolipids to become targets for a wide range of pathogens, particularly in the context of lipid rafts. The so-called “animal glycosphingolipidomes” consist of different glycosphingolipids showing a cellular or tissue-specific structure-based tropism. In a detailed review, Bereznicka et al. describe the role of the microbial lectome-glycolipidome interphase in the recognition, cellular entry, and toxicity of bacterial (*Acinetobacter baumannii, Campylobacter jejuni, Clostridium* sp., *Escherichia coli, Helicobacter pylori, Pseudomonas aeruginosa*, and *Vibrio cholerae*), viral (CMV, HIV, HPV B19, noroviruses and polyomaviruses) and fungal pathogens (Candida spp., *Histoplasma capsulatum, Paracoccidioides brasiliensis*, and *Pneumocystis jirovecii*). The authors comment also on the pathogens-induced evolutionary pressure that has driven host changes in the extracellular glycosphingolipids, producing several carbohydrate-mediated host-pathogens dynamics. They also discuss mounting evidence that would make glycosphingolipids attractive targets for new anti-microbial agents as well as the development of anti-tumor therapies.

The association between ABO blood groups and SARS-CoV-2 infection is still drawing much attention involving different aspects such as pathogenicity, susceptibility and transmission. Studies performed in different clinical settings and geographical regions are providing interesting, but at some time contrasting conclusions, further supporting the need of additional rigorous studies to attain definitive conclusions. In an original research article, Janda et al. evaluated the contribution of ABO/Rh blood group to SARS-CoV-2 susceptibility and symptomatic infection, as well as the role of ABO compatibility between infected and exposed individuals on SARS-CoV-2 transmissibility. This was performed in a German multi-center family cohort study comprising 163 households with 281 children and 355 adults, including seropositive and seronegative individuals. They concluded that individual ABO/Rh blood groups are not independent risk factors of SARS-CoV-2 acquisition and symptomatic infection, neither in children nor in adults. In contrast, although in a different setting, Boukhari et al. report original results of a study comprising French hospital staff and their spouses (a group with high transmission risk) to distinguish between two major pathophysiological mechanisms involved in SARS-CoV-2 transmission, ABO compatibility dependence (or ABO interference) and ABO-dependent intrinsic susceptibility. They conclude that the risk of transmission is much lower in the presence of anti-ABO antibodies, consistent with ABO incompatibility-dependence, suggesting that natural anti-ABO antibodies may provide up to ≈40% protection against COVID-19 transmission. Conversely, ABO dependent intrinsic susceptibility was found unlikely to play a major role. They estimate that at least 14% of possible cases of COVID-19 transmission, at population level in France, were prevented by ABO incompatibility.

The conclusion of this Research Topic further highlights the evolving story of glycans and glycan binding proteins in the modulation of the host-pathogen interphase, pointing to an interesting and growing field of research aimed at both understanding the basic mechanisms that regulate host-pathogen interactions at this interphase, but also translating the accumulated knowledge into improved diagnostics and therapeutics, as well as public health preventive measures in face of emerging infectious diseases.

## Author contributions

All authors listed have made a substantial, direct, and intellectual contribution to the work and approved it for publication.

## Conflict of interest

The authors declare that the research was conducted in the absence of any commercial or financial relationships that could be construed as a potential conflict of interest.

## Publisher's note

All claims expressed in this article are solely those of the authors and do not necessarily represent those of their affiliated organizations, or those of the publisher, the editors and the reviewers. Any product that may be evaluated in this article, or claim that may be made by its manufacturer, is not guaranteed or endorsed by the publisher.
